# Sudden Cardiac Death as the First Manifestation of Arrhythmogenic Right Ventricular Cardiomyopathy in a Young Female Athlete: Postmortem Morphologic Diagnosis

**DOI:** 10.7759/cureus.116999

**Published:** 2026-09-27

**Authors:** Jelena Deronjic, Slobodanka Mitrovic, Marija Radojicic, Milena Ilic, Milica Dimitrijevic Stojanovic, Milos Milosavljevic, Dragan Knezevic, Dalibor Jovanovic

**Affiliations:** 1 Department of Pathology, University Clinical Center Kragujevac, Kragujevac, SRB; 2 Department of Pathology, Faculty of Medical Sciences, University of Kragujevac, Kragujevac, SRB; 3 Center for Vascular Surgery, University Clinical Center Kragujevac, Kragujevac, SRB; 4 Department of Surgery, Faculty of Medical Sciences, University of Kragujevac, Kragujevac, SRB

**Keywords:** arrhythmogenic right ventricular dysplasia, athletes, autopsy, genetic testing, pathology, sudden cardiac death

## Abstract

We report a case of sudden cardiac death in a previously healthy 29-year-old female tennis player who collapsed during a family gathering with no prior cardiovascular symptoms and an unknown family history of sudden death or structural heart disease. A complete autopsy with histopathologic examination revealed a dilated right ventricle with wall thinning to 3 mm and focal transmural replacement of the myocardium by adipose tissue. Microscopically, the right ventricular wall consisted predominantly of adipose tissue, with small residual islands of hypertrophic cardiomyocytes, fibrosis, and sparse inflammatory infiltrates. A lateral left ventricular wall thickness of 15 mm, together with subepicardial fatty infiltration, prompted careful differential diagnostic consideration of athletic remodeling, hypertrophic cardiomyopathy, and biventricular or left-dominant phenotypes of arrhythmogenic cardiomyopathy. Toxicologic analysis was negative. The postmortem gross and histopathologic findings fulfilled major morphologic and histologic criteria and were highly consistent with arrhythmogenic right ventricular cardiomyopathy; however, in the absence of premortem imaging, electrocardiographic, and genetic data, this should be regarded as a postmortem morphologic diagnosis rather than a complete multiparametric diagnosis. This case underscores the diagnostic value of systematic autopsy and histopathologic examination following sudden unexplained death in young athletes, the importance of cautious interpretation of concomitant left ventricular findings, and the need for cardiovascular and genetic evaluation of first-degree relatives.

## Introduction

Arrhythmogenic cardiomyopathy (ACM) is an inherited myocardial disease characterized by progressive fibrofatty or fibrotic replacement of the myocardium, ventricular arrhythmias, and an increased risk of sudden cardiac death [1]. The predominantly right ventricular phenotype is traditionally referred to as arrhythmogenic right ventricular cardiomyopathy (ARVC), historically termed arrhythmogenic right ventricular dysplasia. Although ACM was initially regarded as a disease of the right ventricle, biventricular and left-dominant phenotypes are increasingly recognized [1-3]. The estimated prevalence of the predominantly right ventricular phenotype (i.e., ARVC) is approximately 1:2,000 to 1:5,000, although the true prevalence is difficult to establish because of incomplete penetrance, variable expressivity, and evolving diagnostic criteria [1,2]. Clinical presentation ranges from palpitations, presyncope, syncope, and ventricular tachyarrhythmias to cardiac arrest or sudden cardiac death. Symptoms most commonly emerge during adolescence or adulthood, while disease expression may be influenced by sex, genetic background, and exposure to physical exercise [1,2,4].

Pathogenic variants most commonly involve desmosomal genes such as PKP2, DSP, DSG2, DSC2, and JUP; however, depending on the phenotype, non-desmosomal genes including PLN, TMEM43, DES, FLNC, LMNA, and others may also be involved [1,2,5]. Formal diagnostic criteria for arrhythmogenic right ventricular dysplasia/cardiomyopathy were first proposed in 1994 and were subsequently revised to reflect the broader spectrum of ACM [6].

We present a case of sudden cardiac death in a previously apparently healthy 29-year-old female athlete whose autopsy and histopathologic findings were highly consistent with ARVC. The case is presented to emphasize the importance of postmortem cardiac examination, careful interpretation of left ventricular findings, and family-centered follow-up after sudden unexplained death in a young person. The specific contribution of this case lies in the combination of extensive right ventricular pathological involvement with unexpected left ventricular findings in a female athlete, raising the differential diagnosis of a biventricular phenotype, and in the fact that death occurred at rest rather than during physical exertion in a patient without a reported family history of sudden cardiac death. A purely postmortem diagnosis of ACM is clinically and diagnostically important because it may be the only opportunity to identify a heritable arrhythmogenic disease in cases where premortem symptoms were absent and no antemortem cardiac evaluation was performed; however, such a diagnosis, established without electrocardiographic, imaging, or genetic data, should be regarded as a morphologic diagnosis rather than a complete multiparametric one, a distinction that is addressed in detail in the Discussion.

## Case presentation

A 29-year-old woman who regularly played tennis died suddenly during a family gathering. The information regarding the absence of premortem symptoms and family history was obtained from relatives at the time of the forensic investigation; the exact circumstances immediately preceding the collapse, the estimated time from collapse to death, any resuscitation attempts, and emergency medical or hospital records, if any, were not available to the authors. She had no known history of cardiovascular disease and, according to the available information, had not previously reported symptoms suggestive of arrhythmia, such as palpitations, presyncope, or syncope. There was no reported family history of sudden cardiac death or known structural heart disease. Premortem electrocardiography, echocardiography, cardiac magnetic resonance imaging, ambulatory cardiac rhythm monitoring, and genetic testing were not available to the authors.

A complete autopsy with histopathologic examination was performed. Tissue blocks were obtained from the right and left ventricular free walls at the sites of grossly evident abnormality, without a predetermined systematic protocol encompassing the inflow tract, outflow tract, apex, and diaphragmatic regions of both ventricles. Histologic examination was performed using H&E staining only; no connective tissue-specific stain (e.g., Masson’s trichrome) was performed. The proportion of residual cardiomyocytes and the extent of fibrofatty replacement were assessed qualitatively by visual estimation, without morphometric or other standardized quantitative methods. The right ventricular wall thickness was measured on transverse sections of the free wall, at the site of most pronounced wall thinning, rather than at a single fixed anatomical landmark; a fully standardized measurement site (e.g., a fixed distance from the tricuspid annulus) was not applied. Toxicologic analysis, performed at an external forensic toxicology laboratory, was negative for alcohol, illicit substances, and medications; the specific samples analyzed, analytical methodology, and detection limits were not available to the authors.

On gross examination, the heart weighed 305 g, a value at the upper limit of the reference range for women, without significant cardiomegaly. The right ventricle was dilated, and the right ventricular wall was thinned to 3 mm. Transverse sections showed focal transmural replacement of the myocardial wall by fibrofatty tissue. Myocardial thickness was measured on cross-sections after removal of epicardial adipose tissue, in accordance with standard autopsy practice. The lateral wall of the left ventricle measured 15 mm in thickness, with a small amount of epicardial adipose tissue. A left ventricular wall thickness of 15 mm exceeds the upper limit of the normal range and raises a differential diagnosis that includes hypertrophic cardiomyopathy, athletic remodeling, and a biventricular ACM phenotype; this is discussed in greater detail below. The origin and course of the major coronary arteries (left anterior descending, left circumflex, and right coronary arteries) were unremarkable on gross examination, with no significant atherosclerosis, congenital anomaly, myocardial bridging, or thrombosis identified.

Microscopically, the right ventricular wall was composed predominantly of adipose tissue, with only small islands of residual hypertrophic cardiomyocytes (Figure 1).

**Figure 1:**
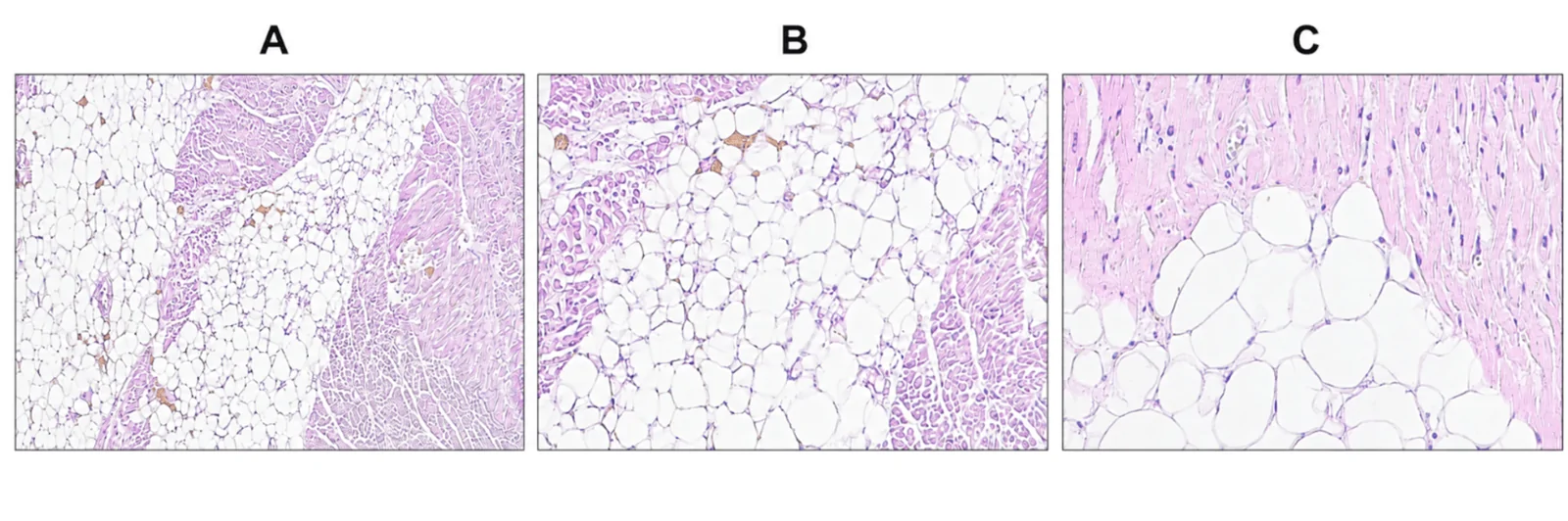
Histopathologic findings in the right ventricular myocardium. (A) Histopathologic section of the right ventricular myocardium (H&E stain, original magnification ×50). A clear interface is seen between extensive fibrofatty tissue (left) and a residual island of more compact, hypertrophic cardiomyocytes (right), with narrow strands of residual myocytes extending through the adipose tissue. (B) A second microscopic field from the right ventricle showing predominant adipose replacement of the myocardium, fibrous bands, and small residual islands of cardiomyocytes; H&E stain, original magnification ×100. (C) Interface between preserved myocardium and fibrofatty replacement at higher magnification (H&E stain, original magnification ×200). Cardiomyocytes on the right show preserved cross-striations and clearly visible nuclei, while large adipocytes immediately adjacent to them replace the myocardial architecture, illustrating the direct interface between residual myocardium and fibrofatty tissue, a pattern compatible with the histopathologic features of ACM. ACM, arrhythmogenic cardiomyopathy

In the left ventricle, myocardial fibers showed an irregular arrangement; individual fibers were short and thick, with focal whorled architecture. The interstitium was focally expanded, with occasional hypercellularity and small clusters of lymphocytes, macrophages, and fibroblasts. Both interstitial fibrosis and replacement-type fibrosis were identified, admixed with fatty infiltration, predominantly in a perivascular distribution, together with marked subepicardial fatty infiltration (Figure 2).

**Figure 2:**
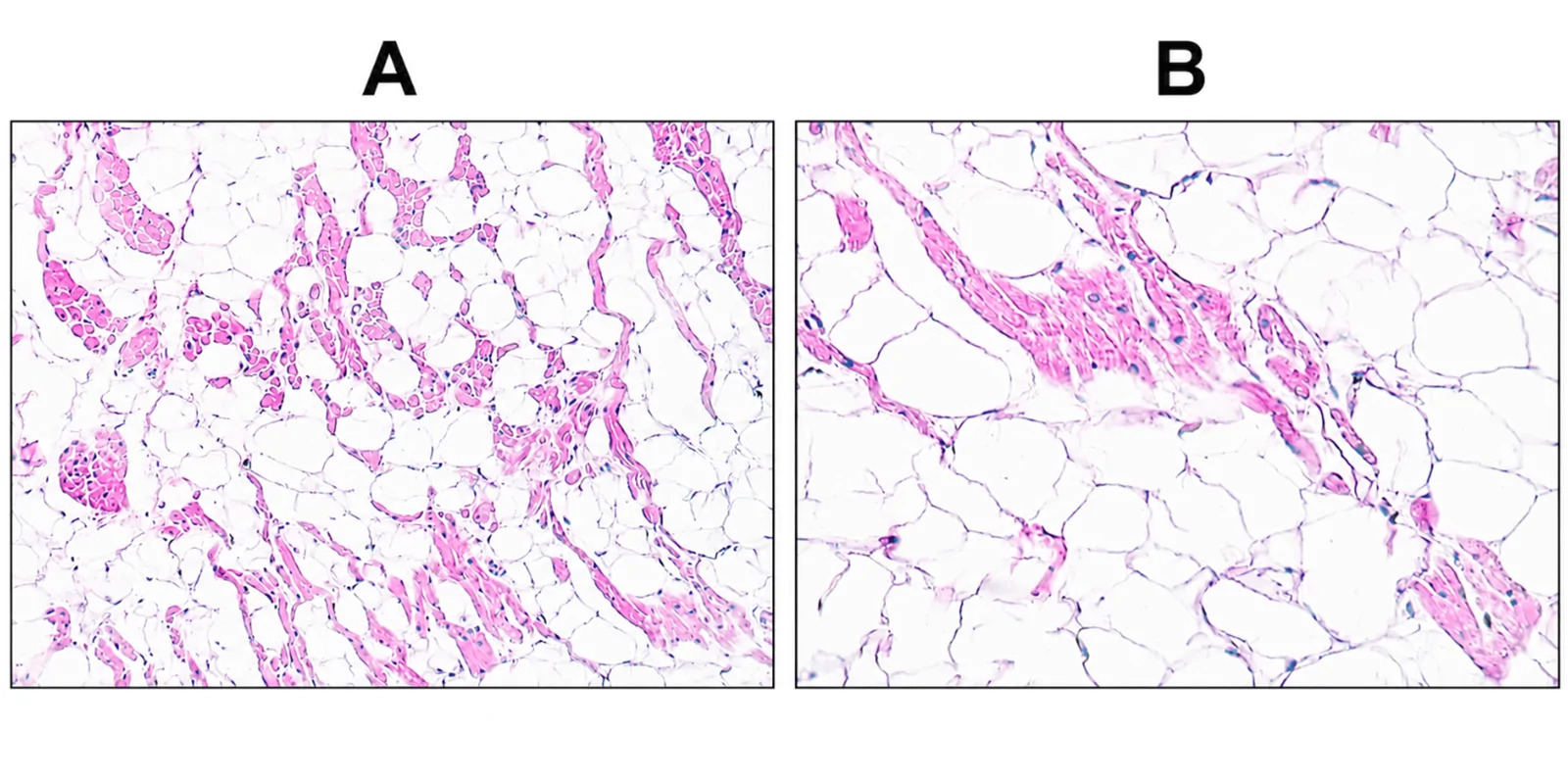
Histopathologic findings in the left ventricular myocardium. (A) Left ventricular myocardium showing extensive fatty infiltration with mature unilocular adipocytes distributed between irregularly arranged cardiomyocyte bundles, with focal cardiomyocyte atrophy and delicate interstitial fibrous septa (H&E stain, original magnification ×100). (B) A second microscopic field from the left ventricle showing extensive fatty infiltration between residual cardiomyocyte bundles, with focal cardiomyocyte atrophy and associated fibrous tissue (H&E stain, original magnification ×200).

No significant histopathologic findings were identified in other organs. A summary of the gross and histopathologic findings is provided in Table 1.

**Table 1:** Key postmortem and histopathologic findings in the present case. ARVC, arrhythmogenic right ventricular cardiomyopathy

Category	Findings
Clinical context	Sudden unexpected death in a 29-year-old female athlete; no known cardiovascular symptoms and no reported family history of sudden cardiac death or structural heart disease.
Toxicology	Negative for alcohol, illicit substances, and medications.
Gross cardiac findings	Heart weight 305 g; dilated right ventricle; right ventricular wall thickness 3 mm; focal transmural replacement of myocardium by fibrofatty tissue; lateral left ventricular wall thickness 15 mm.
Right ventricular histology	Predominant fibrofatty replacement of the right ventricular wall with small residual islands of hypertrophic cardiomyocytes.
Left ventricular/interstitial findings	Irregular, short, and thick myocardial fibers with focal whorled arrangement; sparse inflammatory cells; perivascular fibrotic and fatty replacement; marked subepicardial fatty infiltration.
Other organs	No significant histopathologic abnormalities.
Interpretation	Gross and histopathologic findings highly consistent with ARVC; left ventricular findings require cautious differential diagnostic interpretation.

## Discussion

The central finding in this case was extensive fibrofatty replacement and thinning of the right ventricular myocardium in a young woman who died suddenly without known preceding cardiovascular symptoms. This pattern is highly consistent with ACM. A heart weight of 305 g represents a borderline increase without meeting criteria for cardiomegaly. Progressive fibrofatty replacement and wall atrophy, rather than a marked increase in cardiac mass, have been described in postmortem series of ACM [7]. However, no validated pathological framework correlates heart weight with the extent of fibrofatty replacement, and this finding should not, on its own, be interpreted as an indicator of disease stage. The clinical course is also compatible with a concealed or early phase of the disease, in which structural abnormalities may be limited while the risk of malignant ventricular arrhythmias and sudden cardiac death may nevertheless be substantial [8].

Physical activity is clinically relevant in ACM because endurance and high-intensity training directly contribute to phenotypic expression, more rapid disease progression, and arrhythmic events in genetically predisposed individuals [9,10]. Specifically, tennis is classified among sports with a moderate static and high dynamic component (class IIB), characterized by abrupt high-intensity intervals, sprints, and frequent acceleration and deceleration. This type of exertion causes a substantial acute increase in systolic blood pressure and heart rate, generating marked adrenergic stress. From a pathophysiologic perspective, increased venous return during such exertion leads to pronounced volume loading and mechanical stretch of the geometrically vulnerable and thin right ventricular wall. In individuals with defects of the desmosomal apparatus, this increased mechanical stress accelerates separation of intercalated discs, cardiomyocyte apoptosis, and subsequent reparative fibrofatty replacement, thereby accelerating formation of a substrate for paroxysmal ventricular tachyarrhythmia. Sudden cardiac death may therefore be the first manifestation in young athletes, particularly when the disease was not diagnosed during life [9,10]. In the present case, the absence of documented premortem symptoms or cardiovascular testing illustrates the diagnostic challenge posed by clinically silent disease, a recognized contributor to the underdiagnosis of ACM for which systematic postmortem evaluation has an important corrective role [7]. The patient died at rest during a family gathering rather than during sports activity, which is consistent with the concealed nature of ACM, in which a fatal arrhythmia may occur independently of the timing of physical exertion. However, a quantified history of the patient’s training duration, intensity, competitive level, or frequency was not available in this case. The mechanistic link between exercise exposure and disease expression established in the literature [9,10] should therefore be regarded as general evidence relevant to ACM rather than as an established causal explanation for the substrate observed in this specific patient.

The postmortem gross and histologic findings in the present case are consistent with those described in systematic postmortem series of ACM. Fibrofatty replacement of myocytes associated with ventricular wall atrophy is one of the principal morphologic findings, particularly in the classically described region of the “triangle of dysplasia” [7]. Histologic demonstration of residual cardiomyocytes comprising less than 60% of a section of the right ventricular free wall, together with fibrofatty replacement, constitutes a major histologic criterion under current diagnostic criteria and was morphologically fulfilled in the present case [7,8,11].

The genetic basis of ACM is heterogeneous. Variants in desmosomal genes remain the best-established causes, but contemporary classifications recognize a broader spectrum that includes non-desmosomal genes and phenotypes with right ventricular predominance, biventricular involvement, or left-dominant involvement [1,2,6]. Because genetic testing was not available in this case, a specific genotype cannot be inferred. Nevertheless, a postmortem diagnosis has potential implications for first-degree relatives, in whom clinical and, when appropriate, genetic evaluation should be considered.

Sparse inflammatory infiltrates were identified histologically. Inflammation has been described in some cases of ACM and may overlap clinically or histologically with myocarditis-like episodes [12]. Multifocal aggregates of mononuclear cells, predominantly T lymphocytes, have also been described in postmortem ACM series, but without an established primary causal role [7]. In the present case, the inflammatory component was limited and should not be interpreted as evidence of causality but rather as a possible accompanying finding.

The diagnosis of ACM in living patients is multiparametric and integrates electrocardiography, ambulatory rhythm monitoring, echocardiography, cardiac magnetic resonance imaging, family history, genetic testing, and histopathology when available [13]. Cardiac magnetic resonance imaging may be particularly useful for assessment of ventricular size, function, regional wall-motion abnormalities, and tissue characterization [13]. Endomyocardial biopsy has limited sensitivity for diagnosing ACM because the disease is often segmental and predominantly affects the right ventricular free wall, whereas biopsy specimens are conventionally obtained from the interventricular septum to reduce procedural risk [14]. It is therefore not a universal diagnostic method of choice but may provide useful histologic evidence in selected cases and in the appropriate clinical context [14].

By contrast, in the postmortem setting, systematic autopsy and histopathologic evaluation may provide decisive diagnostic information, particularly when sudden death is unexplained and premortem investigations are unavailable [7]. The extensive fibrofatty replacement of the right ventricle in this case strongly supports ACM as the cause of death. However, the left ventricular findings require careful interpretation. A left ventricular wall thickness of 15 mm exceeds the upper limit of the normal range and raises a differential diagnosis that includes athletic remodeling, hypertrophic cardiomyopathy, biventricular ACM, left-dominant ACM, or nonspecific postmortem changes. In this context, the patient’s sex is relevant to the interpretation of this finding. In female athletes, an echocardiographic or cardiac magnetic resonance-derived LV wall thickness exceeding 12 mm is uncommon and should prompt consideration of hypertrophic cardiomyopathy rather than being readily attributed to physiological athletic remodeling [10,15]. However, this sex-specific threshold was derived from in vivo imaging studies, and the postmortem gross measurement of 15 mm reported in this case is not directly equivalent to an in vivo echocardiographic or cardiac magnetic resonance measurement. Furthermore, LV mass, chamber volumes, body-size indexing, and the overall distribution of hypertrophy (e.g., symmetric versus asymmetric) could not be assessed postmortem. For these reasons, the 15 mm lateral wall thickness observed in this case should not be treated as an independent diagnosis of hypertrophic cardiomyopathy or pathological left ventricular hypertrophy; rather, it should be regarded as one element to be interpreted cautiously alongside the differential diagnostic considerations summarized in Table 2, rather than as a finding that on its own favors structural heart disease over physiologic adaptation to training. In the absence of premortem imaging and genetic testing, these possibilities cannot be completely excluded [2,3,13].

**Table 2:** Differential diagnostic considerations for the left ventricular findings in the present case. ACM, arrhythmogenic cardiomyopathy Table created by the authors based on the findings of the present case and the relevant literature [3,7,9,10,15-17].

Differential diagnosis	Supporting findings	Reasons it was not established as the final diagnosis	Basis
Biventricular ACM	Subepicardial fatty infiltration and fibrosis of the left ventricle, together with typical right ventricular changes.	Reliable phenotypic classification is not possible without premortem imaging and genetic testing.	Not assessable postmortem [3,16,17]
Left-dominant ACM	Left ventricular structural changes may be compatible with this phenotype.	The predominant and diagnostically most compelling findings are present in the right ventricle.	Argues against (relative to biventricular ACM); otherwise not assessable postmortem [3,16,17]
Hypertrophic cardiomyopathy	Left ventricular wall thickness of 15 mm.	Typical myocyte disarray, asymmetric hypertrophy, and clinical/genetic data supporting this diagnosis are lacking.	Argues against (morphology); not assessable (genetic/clinical) [10,15]
Athletic cardiac remodeling	The patient was an active athlete.	Athlete’s heart does not result in extensive fibrofatty replacement and thinning of the right ventricular myocardium.	Argues against [9,10]
Physiologic or nonspecific fatty infiltration	A limited amount of adipose tissue may be physiologic, particularly in a subepicardial location.	Extensive transmural fibrofatty replacement of the right ventricle with myocardial atrophy is not consistent with physiologic fatty infiltration.	Argues against [7]

The subepicardial fatty infiltration of the left ventricle observed in this case warrants particular attention. A subepicardial distribution of fibrosis and fatty replacement in the left ventricle has been described as a characteristic histologic pattern of biventricular and left-dominant ACM phenotypes [3,7], in contrast to the subendocardial pattern typical of ischemic heart disease. In the absence of premortem imaging and genetic analysis, this finding does not permit assignment of a specific phenotype, but it further supports ACM as the diagnostic framework and does not support an ischemic etiology as the primary cause of the left ventricular changes [2,3,13]. The concurrent presence of replacement-type fibrosis, reflecting true myocardial loss, alongside a less specific interstitial component, further supports interpreting the left ventricular changes within the ACM spectrum rather than as a purely reactive or incidental process.

Diagnostic criteria for ACM were first proposed in 1994 and modified in 2010 to improve diagnostic sensitivity and specificity [6,11]. The 2020 Padua criteria and the more recent European Task Force consensus reflect the broader concept of ACM by incorporating right-dominant, biventricular, and left-dominant phenotypes [16,17]. These frameworks are multiparametric diagnostic systems, not purely histopathological scoring systems, and rely on multiple domains, including morphofunctional abnormalities, tissue characterization, repolarization and depolarization changes, arrhythmias, and familial or genetic data. In the present case, several of these domains could not be assessed because the diagnosis was made postmortem. Table 3 lists each relevant diagnostic domain and criterion, the corresponding observation in this case, whether that domain is applicable to postmortem assessment, and the supporting source.

**Table 3:** Application of diagnostic criteria for ACM in the present case. ACM, arrhythmogenic cardiomyopathy; LV, left ventricle; RV, right ventricle Table created by the authors based on the 2010 Task Force Criteria [11], the 2020 Padua criteria [16], and the 2024 European Task Force consensus report [17].

Diagnostic domain	Specific criterion	Pathological/postmortem observation in this case	Applicable postmortem?	Source
Structural/morphofunctional (right ventricle)	Regional/global RV dysfunction and dilation with wall motion abnormality (Task Force); RV structural abnormality (Padua)	Grossly dilated right ventricle with wall thinning to 3 mm	Applicable on gross examination, though without functional/imaging data	[11,16]
Histopathological (right ventricle)	Residual cardiomyocytes <60% by morphometry, with fibrous or fibrofatty replacement in ≥1 sample	Right ventricular free wall composed predominantly of adipose tissue with only small residual islands of cardiomyocytes; estimated qualitatively rather than by morphometry	Applicable, though assessed qualitatively rather than quantitatively in this case	[11,16]
Structural/tissue characterization (left ventricle, Padua)	LV wall motion abnormality or late gadolinium enhancement on cardiac magnetic resonance	Not assessed; only postmortem gross wall thickness and histology available	Not applicable postmortem	[16]
Depolarization/conduction abnormalities	Epsilon wave, QRS prolongation, terminal activation duration	No electrocardiographic recording available	Not applicable postmortem	[11,16,17]
Repolarization abnormalities	T-wave inversion in right or left precordial leads	No electrocardiographic recording available	Not applicable postmortem	[11,16,17]
Arrhythmias	Sustained/nonsustained ventricular tachycardia of RV or LV morphology, ventricular ectopy burden	No ambulatory rhythm monitoring or documented arrhythmia available	Not applicable postmortem	[11,16,17]
Family history/genetics	Confirmed pathogenic variant or family history of ACM/premature sudden death	No family history obtained beyond the reported absence of one; genetic testing not performed	Not applicable in this case	[1,2,6,17]

As summarized in Table 3, only the structural/morphofunctional and histopathological domains of the Task Force and Padua criteria could be assessed on postmortem examination, and the corresponding major criteria in these two domains were fulfilled [11,16]. The depolarization/repolarization, arrhythmic, and family history/genetic domains could not be assessed because of the postmortem context of the diagnosis. Consequently, the diagnosis in this case should be regarded as a postmortem morphologic diagnosis strongly suggestive of ACM, rather than a complete multiparametric diagnosis established according to the full Task Force or Padua criteria.

Therapeutic strategies in diagnosed patients include restriction of high-intensity physical activity, pharmacologic therapy, catheter ablation in selected cases, and implantable cardioverter-defibrillator therapy in individuals at high arrhythmic risk [2,8]. Although these interventions were not possible in this patient, the postmortem diagnosis remains clinically important because it raises suspicion of a heritable cardiomyopathy, which may prompt systematic clinical evaluation of first-degree relatives and, when appropriate, genetic counseling, with the goal of preventing additional sudden deaths within the family [2,7]. This should be regarded as a suspicion-driven recommendation rather than evidence that a pathogenic variant is present in this family.

Compared with previously reported postmortem series of ACM, which predominantly describe isolated right ventricular involvement in male athletes engaged in endurance or high-dynamic sports, the present case adds to the literature by illustrating a combination of severe right ventricular pathological involvement with concurrent, unexpected left ventricular findings in a female athlete engaged in a moderate static/high-dynamic sport, together with the absence of a reported family history of sudden cardiac death. This combination underscores that a biventricular or left-dominant phenotype should be considered even when right ventricular involvement is the diagnostically dominant finding and that a negative family history does not preclude an inherited arrhythmogenic substrate.

Based on the autopsy and histopathologic findings, the underlying disease in this case was strongly suggestive of ACM, based on a postmortem morphologic diagnosis. The immediate mechanism of death is considered to be a malignant ventricular arrhythmia, as no other structural, toxicological, or coronary cause of death was identified; however, this mechanism could not be directly demonstrated, since no electrocardiographic recording was available at the time of death. The cause of death is therefore formulated as sudden cardiac death due to a presumed malignant ventricular arrhythmia in the setting of a postmortem morphologic diagnosis of ARVC.

Limitations

This case report has several limitations. The diagnosis was established solely on the basis of postmortem gross and histopathologic examination, without available premortem electrocardiographic, echocardiographic, cardiac magnetic resonance, or arrhythmologic data. Genetic testing and molecular autopsy were not available, precluding confirmation of a specific genotype and cascade genetic testing of relatives. The cardiac conduction system was not specifically dissected or histologically examined; therefore, gross or microscopic abnormalities involving the conduction system cannot be excluded, and this should be regarded as an additional limitation rather than evidence of a comprehensively excluded structural cause of sudden death. Histologic examination of the coronary arteries was not performed; therefore, microscopic coronary pathology cannot be entirely excluded, although no gross abnormalities were identified. Tissue sampling was not performed according to a predetermined systematic protocol covering all ventricular regions, and connective tissue-specific staining was not performed. The estimate of residual cardiomyocytes below 60% of the right ventricular free wall was based on qualitative visual assessment rather than morphometric quantification; these methodological limitations should be considered when interpreting the histologic findings. In addition, the left ventricular findings could not be fully classified phenotypically because of the absence of clinical and radiologic correlation. Detailed information on the circumstances immediately preceding the collapse, the time interval between collapse and death, resuscitation attempts, and emergency medical or hospital records was not available to the authors, which limits full clinical reconstruction of the terminal event. Nevertheless, the extensive fibrofatty replacement and thinning of the right ventricle, together with the corresponding histopathologic findings, provide a strong morphologic basis for the postmortem diagnosis of ACM.

## Conclusions

ACM may remain clinically silent until a fatal ventricular arrhythmia occurs, and sudden cardiac death may be its first recognized manifestation in young athletes. In this case, systematic autopsy and histopathologic examination demonstrated extensive fibrofatty replacement and thinning of the right ventricle, findings highly consistent with ACM on postmortem morphologic grounds, although a complete multiparametric diagnosis could not be established in the absence of premortem clinical, electrocardiographic, and genetic data.

This case highlights the importance of detailed postmortem cardiac examination after sudden unexplained death in young individuals. It also emphasizes that the associated left ventricular findings, while compatible with a biventricular or left-dominant phenotype, do not definitively establish such a phenotype, and that the available postmortem data do not permit confirmation of a specific genetic substrate in this patient, as well as the importance of family-centered cardiovascular evaluation following a postmortem diagnosis of ACM. First-degree relatives should be referred for standardized clinical cardiovascular evaluation in accordance with current guidelines, as this postmortem diagnosis raises suspicion of a heritable cardiomyopathy rather than providing evidence that a pathogenic variant is present in this family. Genetic counseling and, where appropriate, genetic testing of relatives should be considered in this context, rather than assumed to be automatically indicated or expected to identify a causal variant.
